# Feasibility and Design Factors for Home-Based Pulmonary Rehabilitation of Patients With Chronic Obstructive Pulmonary Disease and Chronic Lung Diseases Based on a People-Object-Environment Framework: Qualitative Interview Study

**DOI:** 10.2196/51150

**Published:** 2024-03-07

**Authors:** Shih-Ying Chien, Alice May-Kuen Wong, Winston Tseng, Han-Chung Hu, Hsiu-Ying Cho

**Affiliations:** 1 Department of Industrial Design Chang Gung University Taoyuan Taiwan; 2 Department of Physical Medicine and Rehabilitation Chang Gung Memorial Hospital Taoyuan Taiwan; 3 Division of Community Health Sciences School of Public Health University of California Berkeley, CA United States; 4 Department of Thoracic Medicine Chang Gung Memorial Hospital Taoyuan Taiwan; 5 Department of Respiratory Therapy Lin-Kou Chang Gung Memorial Hospital Taoyuan Taiwan

**Keywords:** chronic lung diseases, home-based pulmonary rehabilitation, telehealth, remote health care

## Abstract

**Background:**

The feasibility of implementing home-based pulmonary rehabilitation (PR) can be assessed from the perspectives of patients with chronic lung disease and health care professionals involved in PR.

**Objective:**

Through a qualitative inquiry using interviews and the adoption of the people-object-environment framework, this study aims to understand the influences of interpersonal, environmental, and situational factors on the perceptions and considerations of individuals involved in home-based PR for patients with chronic lung disease.

**Methods:**

One-on-one interviews were conducted with 20 patients with chronic lung disease and 20 health care professionals for investigating their attitudes and opinions based on their experiences regarding home-based PR as well as for identifying the key factors affecting the benefits and drawbacks of such therapies. This study further evaluates the feasibility of using digital tools for medical diagnosis and treatment by examining the technology usage of both parties.

**Results:**

The 4 key issues that all participants were the most concerned about were as follows: distance to outpatient medical care, medical efficiency, internet connectivity and equipment, and physical space for diagnosis and treatment. Interviews with patients and health care professionals revealed that the use of technology and internet was perceived differently depending on age and area of residence. Most participants reported that digital tools and internet connectivity had many benefits but still could not solve all the problems; moreover, these same digital tools and network transmission could lead to problems such as information security and digital divide concerns. This study also emphasizes the significant impact of human behavior and thinking on shaping the design of health care interventions and technologies. Understanding user perspectives and experiences is crucial for developing effective solutions for unmet needs.

**Conclusions:**

The results of this study indicate that despite the different perspectives of patients and health care professionals, their considerations of the key issues are very similar. Therefore, the implementation of plans related to telemedicine diagnosis, treatment, or rehabilitation should take the suggestions and considerations of both parties into account as crucial factors for telehealth care design.

## Introduction

As the third leading cause of morbidity and mortality worldwide, chronic obstructive pulmonary disease (COPD) is a significant public health issue [1-4]. In 2019, the number of individuals diagnosed with COPD exceeded 328 million worldwide [5-8]. A significant correlation between physical activity and lung function [9-12] emphasizes the importance of regular exercise for individuals with COPD who require pulmonary rehabilitation (PR) [13-19]. However, patients with COPD often report reluctance to engage in physical activities due to dyspnea, the effects of which include chronic cough, exacerbations, reduced exercise capacity, and impaired quality of life [20-25]. PR is a tailored and comprehensive intervention conducted via a thorough assessment of the patient. In individuals with chronic pulmonary diseases, the primary objective of the pulmonary intervention is to improve not only their overall health but also their psychosocial well-being in the long term [26-28]. Typically, PR programs are customized for personal symptomatic conditions [29-31]; hence, PR interventions entail tailored exercises and educational sessions aimed at enhancing activity tolerance, mitigating symptoms, and augmenting skills that aid in managing chronic respiratory diseases [31,32]. The majority of PR treatments usually require one-on-one sessions and the assistance of a therapist [33-35]. However, the one-on-one care approach is limited due to shortages in health care personnel, elevated work-related stress, and prolonged working hours [36]. Moreover, when the COVID-19 pandemic hit, lockdowns and personnel restrictions forced the interruption of PR for many patients with chronic lung disease, which posed a threat to their lives [37-40].

Due to the COVID-19 pandemic, telehealth has become increasingly attractive owing to its functionality, importance, and prospects [41,42]. In addition to reducing human contact and easing the burden on health care workers, telehealth leverages technology communication and transmission to alleviate the workload of respiratory therapists and improve the accuracy of respiratory rehabilitation records [43-45]. Using telehealth, patients can undergo rehabilitation at home and be monitored remotely by medical personnel [46,47]. Home-based PR can also mitigate the difficulties of outpatient care for patients living in remote areas and those with physical disabilities [48-50]. Furthermore, it can be used as an auxiliary means of physical PR to assist in self-management and precisely modify behavior, thereby reducing hospitalization and medical costs [51,52].

Traditional PR usually relies on one-on-one human monitoring through observation or physiological monitors to examine a patient’s health condition. Remote health care has the advantage of prescribing home-based PR, enabling patients who are unable to leave their homes due to physical conditions such as disability or living in rural areas to partake in rehabilitation programs at home [53,54]. However, there are also many limitations and considerations of remote health care, as follows:

1. Lack of security and limited interpersonal interaction: The safety of patients is the primary concern of clinical physicians [55,56]. The biggest challenge of home rehabilitation is emergency treatment, which has been the main hurdle for remote health care since many years [57]. In addition, remote therapy can only provide limited physical and mental assessments [58,59]. Due to the lack of face-to-face interpersonal interactions, patients may develop loneliness, helplessness, and frustration, which may reduce the effectiveness of treatments and the speed of recovery [60,61].
2. Privacy and security issues: Most remote health care is performed through network transmission. Many clinical physicians believe that network transmission may lead to data leakage or theft of medical records or personal information of patients [62,63].
3. Technological and equipment limitations: The implementation of remote health care requires specific technological equipment such as smartphones or computers with network functions. However, for many remote users or special groups such as older persons, lack of equipment, poor network communication quality, or unfamiliarity with network-related technology hinder utilization [64].
4. Insurance payment limitations: Different regions or countries have different standards for remote health care services. Therefore, many insurance companies do not have a remote health care reimbursement system or only cover specific services [65-68].

Despite its limitations and by taking people, object, and environment into consideration, telemedicine remains a valuable tool for the provision of health care services, especially for patients who have difficulty visiting medical facilities in person or those affected by infectious diseases and related restrictions such as lockdowns and quarantine. Telemedicine enables uninterrupted treatment and continued assistance for patients in their recovery. However, in establishing a home-based PR, it is essential to consider the various environments of participants to effectively maximize the benefits of this medical service.

## Methods

### Ethics Approval

This study was approved by the institutional review board of Chang Gung Memorial Hospital (approval 202200070B0). The participants were patients with chronic lung disease and respiratory health care professionals who had provided written informed consent from both urban and rural areas. Due to the COVID-19 pandemic, all one-on-one interviews were conducted by videoconferencing.

### Participants and Procedures

The 20 patients recruited for the interviews included those who had participated in PR programs and those who had not. During the interviews, the patients provided insights into the implementation of PR programs from a patient-centric standpoint. All interviewees had a medical history of 5 years or more.

The 20 respiratory health care professionals included registered thoracic surgeons, respiratory therapists, physical therapists, and PR specialists. Most of these professionals had experience treating patients with chronic lung disease and had participated in designing exercise prescriptions, patient tracking and monitoring, and disease progression research in PR programs. Furthermore, the majority of the interviewees had treated a specific proportion of patients with chronic lung disease within the past 3 years (Table 1).

**Table 1:** Characteristics of the health care professionals (n=20).

			Values
Gender (male:female)			8:12
Age (years), min-max; mean (SD)			27-65; 46 (11)
Experience in pulmonary rehabilitation (years), mean (SD)			5.2 (8.47)
**Type of health care professional in pulmonary rehabilitation, n (%)**			
	Thoracic surgeons	3 (15)	
	Respiratory therapists	12 (60)	
	Physical therapists	2 (10)	
	Pulmonary rehabilitation specialists	3 (15)	

Prior to the interviews, all participants were required to complete a survey questionnaire, which included demographic information and details of their use of smart devices and the internet. Daily use was defined as regular usage. The patients provided information about their pulmonary disease status, duration of illness, and a self-assessment of their health status (on a 5-point scale ranging from excellent to poor) as well as recalled their activity frequency over the past 7 days. Health care personnel were required to answer questions related to their primary clinical responsibilities. Each participant took part in a 1.5- to 2-hour interview session conducted by the primary author, who was also a clinical researcher and an assistant professor affiliated with the Chang Gung Medical Foundation. In-depth interviews were primarily used to collect the data. After collecting the interview data, all identifiable personal information was removed from the transcripts. The data were then coded, organized, and analyzed using NVivo 12.0 software (Lumivero) for qualitative data analysis. For accurate and detailed data interpretation, the transcripts were provided to the interviewees for review and cross-checked with relevant researchers to confirm the accuracy of data interpretation.

## Results

### Characteristics of the Participants

This study consisted of 40 participants: 20 health care professionals specializing in PR and 20 patients with chronic lung diseases. The background characteristics of the 20 health care professionals are shown in Table 1; nearly 60% (12/20) were respiratory therapists, and the remaining health care professionals were pulmonary surgeons, physical therapists, and rehabilitation physicians. Their mean age was 46 years, and all had more than 3 years of experience in PR and treatment (mean 5.2 years). The background characteristics and activity habits of the 20 patients interviewed are shown in Table 2; the majority of the patients had COPD (12/20, 60%), and 25% (5/20) were lung transplant recipients. The majority of the participants (15/20, 74%) had never participated in a PR program, and 70% (14/20) of the patients rated their physical condition as poor. Regarding exercise over the past week, 65% (13/20) of the patients chose a 10-minute walk as their exercise indicator, followed by strength training (5/20, 25%). Notably, 55% (11/20) of the patients reported preferring to sit rather than stand and to stand rather than move.

**Table 2:** Characteristics of the patients (n=20).

		Values
Gender (male:female)		10:10
Age (years), min-max; mean (SD)		51-85; 68 (9.8)
**Participation in pulmonary rehabilitation programs, n (%)**		
	Yes	5 (26)
	No	15 (74)
**Chronic lung diseases, n (%)**		
	Chronic obstructive pulmonary disease	12 (60)
	Asthma	3 (15)
	Lung transplantation	5 (25)
**Self-assessment of their health, n (%)**		
	Excellent	0 (0)
	Very good	0 (0)
	Good	1 (5)
	Fair	5 (25)
	Poor	14 (70)
**Exercise frequency and quantity over the past week, n (%)**		
	I have engaged in high-intensity strength training, including aerobic exercise, fast cycling, and swimming.	2 (10)
	I have participated in moderate physical activities such as stretching exercises and flexibility training.	5 (25)
	I have walked for at least 10 minutes every day.	13 (65)
**What statement best characterizes my exercise habits? n (%)**		
	Given the option, I will opt to sit rather than stand.	11 (55)
	I frequently require standing but not for the purpose of lifting heavy objects.	5 (25)
	Climbing slopes and stairs is a common necessity for me.	4 (20)
	I often transport heavy objects and engage in manual labor.	0 (0)

### Survey Results

**Figure 1:**
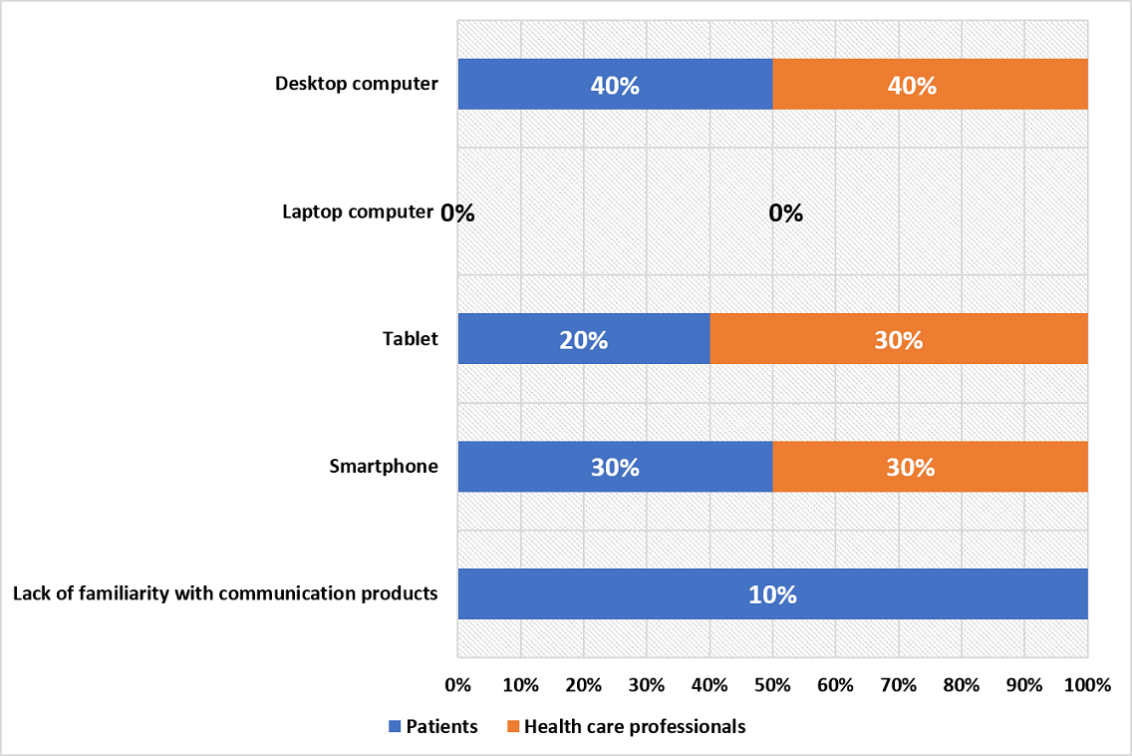
Survey results of the usage of electronic communication devices among patients and health care professionals in this study.

In order to better examine the potential of telehealth, we conducted a survey targeting contemporary electronic communication tools, specifically computers, cellphones, and tablets, which were widely utilized by both patients and health care professionals, as shown in Figure 1. The majority of the patients and health care professionals reported using desktop computers as their most frequently used electronic communication device, constituting the largest proportion at 40% (8/20), followed by smartphones at 30% (6/20). Notably, health care professionals reported a higher usage rate (by 10%) of tablet computers compared to patients. Note that neither group of participants reported habitually using laptops. Overall, the patients were less proficient with technology compared to the health care professionals, which is a crucial determinant in implementing telehealth programs.

### People-Object-Environment Framework

This study adopts the people-object-environment framework as the focal point for the interview investigation to improve the understanding of the feasibility of home-based PR. This study analyzes the advantages and disadvantages of remote PR in the current context, with the aim of bridging the gap between ideal use and reality (Table 3). Activities involving various elements such as individuals, entities, and environmental factors often result in the emergence of diverse concerns among different participants. Through the analysis presented in Table 3, we identified the gaps in home-based rehabilitation services from the perspectives of health care professionals and patients. Subsequently, this facilitated a thorough discussion of potential solutions to meet the needs and expectations of all involved parties. Our research findings reveal that the use of telehealth for home-based PR programs had both advantages and disadvantages. Using a people-object-environment framework to analyze the results, we describe 4 dimensions: reduced time and transportation constraints to access medical care; improved medical efficiency; changes in equipment, network, and physical space; and information transmission security, about which health care professionals particularly raised concerns in telehealth.

**Table 3:** Analysis of the pros and cons of telerehabilitation with the people-object-environment framework.

		People (patient)	Object (patient)	Environment (patient)
**People (health care professionals)**				
	Pros	Promoting health care access in remote areas: Telehealth facilitates convenient health care services, which enhance medical care for patients in remote areas and promote community health. Boosting patient involvement: Telehealth enables interactions with health care professionals via web-based platforms, providing medical information and guidance as well as fostering active engagement of patients in their own health management.	Improving health care resource allocation: Telehealth enables physicians to diagnose and treat patients across different geographical areas, alleviating shortages in local health care resources and enhancing the efficiency of health care resource allocation.	Reducing health care burden: Telehealth reduces the health care burden for long-term patients or those requiring regular follow-ups; this minimizes the time and effort associated with transportation and waiting as well as provides cost-effective health care options. Decreasing cross-infection risks: Telehealth minimizes contact between patients and health care professionals, thereby lowering the risk of cross-infection and promoting the health and safety of both health care professionals and patients.
	Cons	Bridging communication barriers: Telehealth reduces physical interactions and social contact between patients and physicians, which may have long-term effects on patients’ psychological and social well-being.	Operational and communication barriers: Older or technologically inexperienced patients may encounter difficulties in understanding instructions from remote health care professionals through telehealth.	Environmental limitations: Home environments often impose spatial constraints that may limit various rehabilitation, diagnostic, and treatment activities.
**Object (health care professionals)**				
	Pros	Advantages of telehealth: Telehealth provides a convenient health care model, particularly beneficial for regions facing constraints related to time, geographical location, and transportation. The utilization of basic computer equipment enables the provision of medical consultations, making health care more accessible and efficient for patients in such areas.	Wireless transmission: Wireless transmission significantly reduces the workload of health care professionals and makes health care services more efficient by transitioning from a one-on-one service to a one-to-many format.	Digital health care infrastructure: Telehealth accelerates the transmission and exchange speed of health care information, leading to improved overall health care efficiency.
	Cons	Digital divide: Individuals who are from lower socioeconomic backgrounds or have limited access to digital resources may face barriers to participation in telehealth due to the lack of appropriate technological equipment or internet connectivity. This highlights inequalities in the distribution of health care resources. Technological dependency: Users with limited technological skills or resources may encounter difficulties in operating telehealth, which requires adequate knowledge of technology, suitable equipment, and stable internet connectivity. Health care quality and patient experience: Although telehealth provides convenient remote health care options for certain diseases or conditions, in-person consultations or measurements from medical instruments may offer more accurate health care services.	Disparity in health care resources between urban and rural areas: Despite the convenience of telehealth for remote consultations, operational difficulties may still exist for areas lacking proper equipment.	Equipment and infrastructure requirements: Telehealth relies on high-speed internet and appropriate equipment, which can still pose challenges in certain rural areas.
**Environment (health care professionals)**				
	Pros	Expansion of health care service areas: Through telehealth, physicians can diagnose and treat patients remotely without being limited by geographical location while providing real-time medical services. Expansion of professional scope: Telehealth enables physicians to engage in remote meetings and collaborations with other health care experts, enhancing medical efficiency. Enhancement of diagnosis and treatment efficiency: Telehealth reduces time and space limitations between physicians and patients, improving the efficiency of the overall health care services. Increased convenience: Telehealth offers patients greater convenience, particularly for those residing in remote areas or facing mobility challenges, thereby reducing the time and costs associated with hospital visits.	Enhancement of diagnostic and treatment capabilities: Through remote imaging and information sharing, physicians can access additional support and assistance, which improve diagnostic accuracy and treatment outcomes. Improvement of health care resource utilization: Telehealth aids physicians in managing and allocating regional health care resources more effectively, enhancing utilization efficiency and reducing unnecessary health care costs.	Reducing reliance on physical space: Telehealth reduces the need for physical space such as clinics and hospitals, thereby lowering costs and burdens associated with facilities and resources for health care institutions.
	Cons	Lack of physical contact: Telehealth may not provide opportunities for face-to-face contact with patients, which can make it challenging for physicians to conduct comprehensive physical examinations or assessments. Limitations in comprehensive treatment: Some diagnoses and treatments may require physical contact and assistance from specific equipment, which cannot necessarily be substituted by telehealth.	Technical requirements: The use of telehealth requires stable internet connectivity and appropriate device support, which may be challenging for users who are not familiar with technology. Medical responsibility and risk management: Telehealth may involve issues of medical responsibility and risk management, such as misdiagnosis, treatment errors, or incomplete medical records, which may result in medical disputes and litigation. Physicians and health care institutions need to ensure compliance with relevant medical responsibility and risk management principles in telehealth as well as maintain a high level of medical practice.	Health care security and privacy risks: The use of telehealth may involve health care security and digital privacy risks such as patient identity verification and medical record protection. Physicians should exercise caution in handling such issues.

### Dimensions

#### Dimension 1: Distance, Time, and Transportation Issues

Both patients and health care professionals acknowledged the significant benefits of telehealth in addressing the challenges of distance in accessing medical care. Reductions in travel and wait times due to telehealth allowed patients to actively participate in their health care decision-making through web-based platforms. The digitization of medical records for better disease management was also facilitated. For residents in remote areas, telehealth eliminated geographical barriers to health care, promoted more efficient allocation of medical resources, and prevented the closure of regional hospitals and the “medical deserts” phenomenon. Nevertheless, health care professionals identified potential risks and quality-of-care issues associated with telehealth. Due to the absence of physical interactions, physicians were limited to relying on surface-level symptoms for diagnosis. Comprehensive physiological examinations and physical evaluations were also limited due to the lack of suitable equipment, which could in fact jeopardize the safety of critically ill patients who require urgent or emergency treatment. Moreover, prolonged social isolation resulting from telehealth may have adverse effects on patients’ psychological and social well-being.

#### Dimension 2: Enhancing Medical Efficiency Through the Use of Telehealth

Health care professionals described that the popularization of telehealth was due to its advantages in improving the efficiency of disseminating medical information. Through online platforms, health care professionals can access patients’ medical history instantaneously and collaborate to provide optimized treatment. This approach reduced constraints on patients’ time and space, expanded the scope of medical services, reduced the workload of health care professionals, and transformed the traditional one-on-one respiratory treatment mode into a one-to-many model. Health care professionals also noted that the advantages of data and imaging arising from telehealth actually improved diagnostic and treatment efficiency, which could as a result achieve precision medicine. However, health care professionals also raised concerns about telehealth. The mode of transmitting medical information through data still harbored many risks and considerations such as diagnostic and treatment errors. Additionally, injuries (such as falls or respiratory distress) that could occur during treatment raised issues related to medical responsibility and risk management. Therefore, handling patient identity verification and medical records with caution when administering telehealth is crucial in order to safeguard patient privacy during medical treatments.

#### Dimension 3: Leveraging Internet Connectivity and Device-Based Solutions for Rehabilitation and Health Monitoring

Patients and health care professionals both identified that digital health care had the potential to significantly reduce medical wait times and enhance efficiency, which complemented the services of regional hospitals, lowered the medical burden of chronic patients, and eliminated limitations due to transportation, geography, and time. However, many challenges still remain with the use of digital tools for rehabilitation and monitoring systems. Most patients who require PR are older, aged ≥65 years, and unfamiliar with digital devices and networks, and they often lack the knowledge and understanding of how to install and configure such devices and applications. In addition, remote areas lack stable networks, technology, and equipment. This digital divide inhibits a subset of patients in certain areas from fully utilizing relevant medical services, highlighting the problem of an uneven distribution of medical resources.

#### Dimension 4: Advantages and Disadvantages of Converting Medical Spaces

The changing medical environment has brought many advantages to patients and health care institutions through telehealth, particularly during the COVID-19 pandemic, by reducing hospital-acquired infections and patients’ reliance on medical space and resources, thereby alleviating the burden of health care costs. However, as previously mentioned, some health care professionals reported that not all diagnoses and treatments could be properly conducted remotely due to the availability or operation of equipment and the limitation of patients’ home space and environment, which restrict the implementation of many treatment regimens. All the 4 dimensions supported by quotes from patients and health care professionals are shown in Table 4.

**Table 4:** Verbatim quotes supporting the main dimensions by patients and health care professionals.

Dimensions		Patient	Health care professional
**Dimension 1: Distance to outpatient care**			
	Positive	…*During the pandemic, being able to have online consultations reduced a lot of my stress. I heard many of my friends around me were infected while at hospital.* [Female, 48 years old]	…*Telemedicine has reduced a lot of transport-related issues. Through online connections, we can access all of the patient’s data and make more accurate assessments.* [HCP^a^ #12]
	Negative	…*I live in a very rural area where there are no taxis, so every time I see a doctor, I have to take four different buses. The journey alone takes me over three hours, so I avoid seeing a doctor if I can help it.* [Female, 64 years old]	…*To be honest, not every patient is suitable for telemedicine. For example, older patients may have difficulty understanding what I ask them to do. Also, some patients’ conditions cannot be determined solely by questioning and require examination using medical instruments and devices, so it is difficult for me to make a diagnosis without a proper examination.* [HCP #8]
**Dimension 2: Medical efficiency**			
	Positive	…*Because I get breathless when I walk, I try not to go out if I don’t have to. I’m also afraid of falling when I go out, and I don’t want to bother my children. So if I can see a doctor through a computer, I prefer that.* [Male, 66 years old]	…*The biggest advantage of telehealth is saving a lot of time and manpower. Of course, this refers to medical work that is more repetitive and lower risk. But I hope that in the future, online systems will have warning functions that can quickly let me know which patient has an issue that needs special attention.* [HCP #3]
	Negative	…*I would rather see a real doctor. Just talking on the phone doesn’t give me a feeling that I’ve really seen a doctor.* [Female, 72 years old]	…*To be honest, although the internet is convenient, I feel that its effectiveness is sometimes limited. Perhaps respiratory therapy needs to be divided into stages, and not every stage is suitable for being done at home. It may need to be classified/graded.* [HCP #17]
**Dimension 3: Internet connectivity and equipment**			
	Positive	…*Every time I go out to see a doctor, I’m always in a rush and get so nervous that I forget to ask the doctor any questions. By seeing the doctor through a computer, I have more time to chat with the doctor.* [Female, 70 years old]	…*The internet connection is very convenient. As long as the health insurance card is inserted, all the patient’s information can be accessed. Telehealth has not only changed a patient’s medical treatment mode but also prevented many regional hospitals from closing down.* [HCP #11]
	Negative	…*To be honest, I don’t really understand the internet. If no one helps me set it up, I won’t know how to see a doctor online. And if the doctor doesn’t see me, how will they know what’s wrong with me?* [Male, 76 years old]	…*Sometimes, the reason why I cannot wait for a patient is because the foreign caregiver has not set up the computer properly. When communicating with the patient through the computer, sometimes the elderly cannot understand, and it is also difficult to communicate with the caregiver. If I were there in person, I could still teach them how to do it.* [HCP #8]
**Dimension 4: Space for diagnosis and treatment**			
	Positive	…*I am old and unable to move around easily. It would be best for me to see the doctor at home.* [Male, 86 years old]	…*A patient receiving online medical care at home will require much less space for us, such as waiting rooms and registration areas. It will also significantly reduce the demand and burden on staff.* [HCP #1]
	Negative	…*There are many things that I cannot do at home. I need to have my blood pressure measured, but there is no one to help me at home. Also, I like to chat with people, but at home, there’s only me.* [Female, 70 years old]	…*Online medical care now is quite good, with many complete functions such as registration, appointment progress, and electronic medical records. However, I personally have reservations about having many medical records stored in the cloud, as there is no absolute security. Also, if a patient falls at home, how to allocate responsibility and the risks involved are also concerns.* [HCP #14]

^a^HCP: health care professional.

## Discussion

This study explores the perspectives and barriers of respiratory health care professionals and patients toward telehealth for rehabilitation for respiratory diseases. Based on the participant interviews, the use of home-based telerehabilitation for patients with lung diseases was perceived to have both advantages and disadvantages, which could be categorized under 4 domains: location, digital technology, internet connectivity, and physical space requirements. Unlike previous research [69], we adopted the people-object-environment framework and interviewed patients as well as health care professionals, with the aim of obtaining feedback from all participants in the same context. The 4 aspects mentioned above were found to be the most important concerns for health care institutions and patients.

Previous studies have reported that distance to outpatient care has a profound impact on chronic patients; in other words, the farther away from home, the lower is a patient’s willingness to seek medical care [70]. However, Bhatt et al [71,72] highlighted that patients often exhibit a reluctance to engage in PR, regardless of proximity, for several reasons. Both groups of interviewees in our study reported that the provision of alternative options would reduce the number of hospital visits, which would benefit patients and health care institutions. Although digital health care has its limitations, leveraging the internet to expand regional hospital services is not only beneficial to the public but also makes medical services more effective [73]. In special circumstances such as the outbreak of a pandemic, issues such as patients being unable to attend in-person treatments cannot be ignored. It is undeniable that digital health care, in particular, spawns numerous benefits in such situations. However, there are still limitations to digital health care for people (doctors or the public), equipment and network, and the environment (urban or rural). For example, most older patients feel that only consultations in person with doctors generate the feeling of being treated. Furthermore, without physical examinations, it could be difficult for doctors to diagnose the cause of symptoms. Nonetheless, digital technology remains a good choice for patients with respiratory disease who do not want to venture outside or exercise; however, not every patient’s home is equipped with remote medical devices or equipment, especially in rural areas [74]. Most patients with respiratory disease are also older, and without caregiver assistance, operating such devices can be difficult. In addition, due to limited professional knowledge, equipment, network, and living space, home care cannot replace all hospital diagnoses and rehabilitation. Therefore, in consideration of the findings from our study and a previous study [75], the implementation of hierarchical medical care requires that patients first undergo video consultations and then be referred to nearby medical institutions for appropriate treatment based on the severity of their condition. Patients can be referred to a larger medical center when necessary for treatment through an electronic referral platform between institutions. This approach not only effectively improves the utilization efficiency of medical resources but also significantly reduces medical expenses and transportation costs for patients.

This study has some limitations. First, as our study was conducted in a specific health care institution, our findings may not be generalizable to other regions or institutions. Second, most patients had poor health conditions and no prior experience with PR; thus, they relied only on limited experience and information. Lastly, the majority of the participants were older, which may have influenced their responses regarding computer use or internet issues. Additionally, health care service needs likely vary between urban and rural areas, and our study does not distinguish between the challenges and differences in home-based PR between these 2 types of areas. Future research should consider this aspect in their study design.

Most study participants reported that telehealth could greatly benefit patients with chronic pulmonary diseases; however, these benefits were not without limitations. Reflections on these limitations by patients or health care professionals revealed that telehealth is not suitable for all patients. For example, diagnosis and treatment via telehealth can only accomplish certain tasks and merely serve as a tool for preliminary diagnostic assessments. Nonetheless, preliminary assessments can determine whether a referral to a regional hospital or a large teaching hospital is necessary. This classification and referral system will also be applicable to rehabilitation therapy. Not all patients are suitable for home-based PR, considering patient safety, the required space and equipment, or the need for further precision testing, among other factors. In addition, although telehealth brought many conveniences to patients and health care professionals, both parties still faced significant psychological pressure. Patients noted that digital medicine lacked warmth, and they tended to prefer human care, while physicians had doubts about medical decision-making without the ability to perform physical examinations. The degree of control over digital technology was also an issue. Both parties lacked confidence that effective treatment could be achieved solely through the internet. Even though digital care has the advantage of long-term patient monitoring, some patients were unfamiliar with internet devices, and health care professionals were concerned that patients may not always respond correctly to instructions. Moreover, patients often neglect their physician’s advice (such as following prescribed exercise schedules) due to lack of motivation and the need to physically meet with the physician. Both groups of study participants indicated that significant improvements in telerehabilitation technology were still needed, particularly for patients in rural areas or those who were older and living alone, who require more support and services.

## Abbreviations

COPD: chronic obstructive pulmonary disease
PR: pulmonary rehabilitation

## References

[1] Viegi Giovanni, Maio Sara, Pistelli Francesco, Baldacci Sandra, Carrozzi Laura (2006). Epidemiology of chronic obstructive pulmonary disease: health effects of air pollution. Respirology.

[2] Cukic V, Lovre V, Dragisic D, Ustamujic A (2012). Asthma and chronic obstructive pulmonary disease (COPD) - differences and similarities. Mater Sociomed.

[3] Chapman KR, Mannino D M, Soriano J B, Vermeire P A, Buist A S, Thun M J, Connell C, Jemal A, Lee T A, Miravitlles M, Aldington S, Beasley R (2006). Epidemiology and costs of chronic obstructive pulmonary disease. Eur Respir J.

[4] Pauwels R (2001). Global initiative for chronic obstructive lung diseases (GOLD): time to act. Eur Respir J.

[5] Whittaker Brown S, Braman S (2020). Recent advances in the management of acute exacerbations of chronic obstructive pulmonary disease. Med Clin North Am.

[6] López-Campos José Luis, Tan Wan, Soriano Joan B (2016). Global burden of COPD. Respirology.

[7] Nguyen HT, Collins PF, Pavey TG, Nguyen NV, Pham TD, Gallegos DL (2019). Nutritional status, dietary intake, and health-related quality of life in outpatients with COPD. Int J Chron Obstruct Pulmon Dis.

[8] GBD 2015 Chronic Respiratory Disease Collaborators (2017). Global, regional, and national deaths, prevalence, disability-adjusted life years, and years lived with disability for chronic obstructive pulmonary disease and asthma, 1990-2015: a systematic analysis for the Global Burden of Disease Study 2015. Lancet Respir Med.

[9] Hartman JE, Boezen HM, de Greef MH, Bossenbroek L, ten Hacken NH (2010). Consequences of physical inactivity in chronic obstructive pulmonary disease. Expert Rev Respir Med.

[10] Pitta F, Troosters T, Spruit MA, Probst VS, Decramer M, Gosselink R (2005). Characteristics of physical activities in daily life in chronic obstructive pulmonary disease. Am J Respir Crit Care Med.

[11] Han Y, Heo Y, Hong Y, Kwon SO, Kim WJ (2019). Correlation between physical activity and lung function in dusty areas: results from the chronic obstructive pulmonary disease in dusty areas (CODA) cohort. Tuberc Respir Dis (Seoul).

[12] Garcia-Aymerich J, Serra I, Gómez Federico P, Farrero E, Balcells E, Rodríguez Diego A, de Batlle J, Gimeno E, Donaire-Gonzalez D, Orozco-Levi M, Sauleda J, Gea J, Rodriguez-Roisin R, Roca J, Agustí Àlvar G, Antó Josep M, PhenotypeCourse of COPD (PAC-COPD) Study Group (2009). Physical activity and clinical and functional status in COPD. Chest.

[13] Jenkins Sue, Hill Kylie, Cecins Nola M (2010). State of the art: how to set up a pulmonary rehabilitation program. Respirology.

[14] Bourbeau J (2010). Making pulmonary rehabilitation a success in COPD. Swiss Med Wkly.

[15] Spruit MA, Pitta F, McAuley E, ZuWallack RL, Nici L (2015). Pulmonary rehabilitation and physical activity in patients with chronic obstructive pulmonary disease. Am J Respir Crit Care Med.

[16] Williams V, Bruton A, Ellis-Hill C, McPherson K (2010). The effect of pulmonary rehabilitation on perceptions of breathlessness and activity in COPD patients: a qualitative study. Prim Care Respir J.

[17] Donner C, Muir J (1997). Selection criteria and programmes for pulmonary rehabilitation in COPD patients. Rehabilitation and Chronic Care Scientific Group of the European Respiratory Society. Eur Respir J.

[18] Fishman AP (1994). Pulmonary rehabilitation research. Am J Respir Crit Care Med.

[19] Troosters T, Gosselink R, Janssens W, Decramer M (2010). Exercise training and pulmonary rehabilitation: new insights and remaining challenges. Eur Respir Rev.

[20] Dürr Selina, Zogg S, Miedinger D, Steveling EH, Maier S, Leuppi JD (2014). Daily physical activity, functional capacity and quality of life in patients with COPD. COPD.

[21] José Anderson, Dal Corso S (2016). Inpatient rehabilitation improves functional capacity, peripheral muscle strength and quality of life in patients with community-acquired pneumonia: a randomised trial. J Physiother.

[22] Holland AE, Wadell K, Spruit MA (2013). How to adapt the pulmonary rehabilitation programme to patients with chronic respiratory disease other than COPD. Eur Respir Rev.

[23] Blumenthal JA, Keefe FJ, Babyak MA, Fenwick CV, Johnson JM, Stott K, Funk RK, McAdams MJ, Palmer S, Martinu T, Baucom D, Diaz PT, Emery CF (2009). Caregiver-assisted coping skills training for patients with COPD: background, design, and methodological issues for the INSPIRE-II study. Clin Trials.

[24] Maurer J, Rebbapragada V, Borson S, Goldstein R, Kunik ME, Yohannes AM, Hanania NA, ACCP Workshop Panel on AnxietyDepression in COPD (2008). Anxiety and depression in COPD: current understanding, unanswered questions, and research needs. Chest.

[25] Kessler R, Partridge MR, Miravitlles M, Cazzola M, Vogelmeier C, Leynaud D, Ostinelli J (2011). Symptom variability in patients with severe COPD: a pan-European cross-sectional study. Eur Respir J.

[26] Spruit MA, Singh SJ, Garvey C, ZuWallack R, Nici L, Rochester C, Hill K, Holland AE, Lareau SC, Man WD, Pitta F, Sewell L, Raskin J, Bourbeau J, Crouch R, Franssen FME, Casaburi R, Vercoulen JH, Vogiatzis I, Gosselink R, Clini EM, Effing TW, Maltais F, van der Palen J, Troosters T, Janssen DJA, Collins E, Garcia-Aymerich J, Brooks D, Fahy BF, Puhan MA, Hoogendoorn M, Garrod R, Schols AMWJ, Carlin B, Benzo R, Meek P, Morgan M, Rutten-van Mölken Maureen P M H, Ries AL, Make B, Goldstein RS, Dowson CA, Brozek JL, Donner CF, Wouters EFM, ATS/ERS Task Force on Pulmonary Rehabilitation (2013). An official American Thoracic Society/European Respiratory Society statement: key concepts and advances in pulmonary rehabilitation. Am J Respir Crit Care Med.

[27] Bolton CE, Bevan-Smith EF, Blakey JD, Crowe P, Elkin SL, Garrod R, Greening NJ, Heslop K, Hull JH, Man WD, Morgan MD, Proud D, Roberts CM, Sewell L, Singh SJ, Walker PP, Walmsley S, British Thoracic Society Pulmonary Rehabilitation Guideline Development Group, British Thoracic Society Standards of Care Committee (2013). British Thoracic Society guideline on pulmonary rehabilitation in adults. Thorax.

[28] Donner C, Ambrosino N, Goldstein RS (2020). Pulmonary Rehabilitation.

[29] Billings PR, Kohn MA, de Cuevas M, Beckwith J, Alper JS, Natowicz MR (1992). Discrimination as a consequence of genetic testing. Am J Hum Genet.

[30] Olivier C, Grosbois J, Cortot AB, Peres S, Heron C, Delourme J, Gierczynski M, Hoorelbeke A, Scherpereel A, Le Rouzic O (2018). Real-life feasibility of home-based pulmonary rehabilitation in chemotherapy-treated patients with thoracic cancers: a pilot study. BMC Cancer.

[31] Ryan P, Sawin KJ (2009). The individual and family self-management theory: background and perspectives on context, process, and outcomes. Nurs Outlook.

[32] Sohanpal R (2015). Understanding the reasons for non participation in self-management interventions amongst patients with chronic conditions: addressing and increasing opportunities for patients with advanced chronic obstructive pulmonary disease to access self-management [theses]. Queen Mary University of London.

[33] Hall C, Nici L, Sood S, ZuWallack R, Castro M (2017). Nonpharmacologic therapy for severe persistent asthma. J Allergy Clin Immunol Pract.

[34] Ruppel GL, Enright PL (2012). Pulmonary function testing. Respir Care.

[35] Shah S, Nasb Mohammad, Lu Min, Huang Liangjiang, Wang Yizhao, Chen Hong (2020). Scaling the need, benefits, and risks associated with COVID-19 acute and postacute care rehabilitation: a review. Rehabil Res Pract.

[36] Czaja S, Sharit J (2012). Human resource management. Designing Training and Instructional Programs for Older Adults.

[37] Moubarak S, Merheb D, Basbous L, Chamseddine N, Bou Zerdan M, Assi HI (2022). COVID-19 and lung cancer: update on the latest screening, diagnosis, management and challenges. J Int Med Res.

[38] Tabassum T, Rahman A, Araf Y, Ullah MA, Hosen MJ (2022). Management of asthma patients during the COVID-19 pandemic: pathophysiological considerations to address the challenges. Beni Suef Univ J Basic Appl Sci.

[39] Gharibo C, Sharma Amit, Soin Amol, Shah Shalini, Diwan Sudhir, Buenaventura Ricardo, Nampiaparampil Devi E, Aydin Steve, Bakshi Sanjay, Abdi Salahadin, Jha Sachin Sunny, Cordner Harold, Kaye Alan D, Abd-Elsayed Alaa, Candido Kenneth D, Knezevic Nebojsa Nick, Atluri Sairam, Wargo Bradley W, Sanapati Mahendra R, Datta Sukdeb, Hirsch Joshua A, Manchikanti Laxmaiah, Rajput Kartic (2020). Triaging interventional pain procedures during COVID-19 or related elective surgery restrictions: evidence-informed guidance from the American Society of Interventional Pain Physicians (ASIPP). Pain Physician.

[40] Coker M, Folayan MO, Michelow IC, Oladokun RE, Torbunde N, Sam-Agudu NA (2021). Things must not fall apart: the ripple effects of the COVID-19 pandemic on children in sub-Saharan Africa. Pediatr Res.

[41] Bokolo AJ (2021). Exploring the adoption of telemedicine and virtual software for care of outpatients during and after COVID-19 pandemic. Ir J Med Sci.

[42] Lukas H, Xu C, Yu Y, Gao W (2020). Emerging telemedicine tools for remote COVID-19 diagnosis, monitoring, and management. ACS Nano.

[43] Tan JY, Conceicao EP, Wee LE, Sim XYJ, Venkatachalam I (2021). COVID-19 public health measures: a reduction in hospital admissions for COPD exacerbations. Thorax.

[44] Sharma P, Pandey AK, Bhattacharyya DK (2021). Determining crucial genes associated with COVID-19 based on COPD findings. Comput Biol Med.

[45] Radzikowska U, Ding M, Tan G, Zhakparov D, Peng Y, Wawrzyniak P, Wang M, Li S, Morita H, Altunbulakli C, Reiger M, Neumann AU, Lunjani N, Traidl-Hoffmann Claudia, Nadeau KC, O'Mahony Liam, Akdis C, Sokolowska M (2020). Distribution of ACE2, CD147, CD26, and other SARS-CoV-2 associated molecules in tissues and immune cells in health and in asthma, COPD, obesity, hypertension, and COVID-19 risk factors. Allergy.

[46] Sakai T, Hoshino C, Yamaguchi R, Hirao M, Nakahara R, Okawa A (2020). Remote rehabilitation for patients with COVID-19. J Rehabil Med.

[47] Wickerson L, Helm D, Gottesman C, Rozenberg D, Singer LG, Keshavjee S, Sidhu A (2021). Telerehabilitation for lung transplant candidates and recipients during the COVID-19 pandemic: program evaluation. JMIR Mhealth Uhealth.

[48] Brundisini F, Giacomini M, DeJean D, Vanstone M, Winsor S, Smith A (2013). Chronic disease patients' experiences with accessing health care in rural and remote areas: a systematic review and qualitative meta-synthesis. Ont Health Technol Assess Ser.

[49] Russell TG, Buttrum P, Wootton R, Jull GA (2011). Internet-based outpatient telerehabilitation for patients following total knee arthroplasty. Journal of Bone and Joint Surgery.

[50] Roberts P (2004). Staffing an empty schoolhouse: attracting and retaining teachers in rural, remote and isolated communities. ERIC.

[51] Fitzner K, Moss G (2013). Telehealth--an effective delivery method for diabetes self-management education?. Popul Health Manag.

[52] Martin MA, Catrambone CD, Kee RA, Evans AT, Sharp LK, Lyttle C, Rucker-Whitaker C, Weiss KB, Shannon JJ, Chicago Initiative to Raise Asthma Health Equity Investigative Team (2009). Improving asthma self-efficacy: developing and testing a pilot community-based asthma intervention for African American adults. J Allergy Clin Immunol.

[53] Brennan D, Mawson Sue, Brownsell Simon (2009). Telerehabilitation: enabling the remote delivery of healthcare, rehabilitation, and self management. Stud Health Technol Inform.

[54] Dew A, Bulkeley K, Veitch C, Bundy A, Gallego G, Lincoln M, Brentnall J, Griffiths S (2013). Addressing the barriers to accessing therapy services in rural and remote areas. Disabil Rehabil.

[55] Hasson S, Waissengrin Barliz, Shachar Eliya, Hodruj Marah, Fayngor Rochelle, Brezis Mirika, Nikolaevski-Berlin Alla, Pelles Sharon, Safra Tamar, Geva Ravit, Wolf Ido (2021). Rapid implementation of telemedicine during the COVID-19 pandemic: perspectives and preferences of patients with cancer. Oncologist.

[56] Al Ameen M, Liu J, Kwak K (2012). Security and privacy issues in wireless sensor networks for healthcare applications. J Med Syst.

[57] Patel S, Park H, Bonato P, Chan L, Rodgers M (2012). A review of wearable sensors and systems with application in rehabilitation. J Neuroeng Rehabil.

[58] Barnett P, Goulding Lucy, Casetta Cecilia, Jordan Harriet, Sheridan-Rains Luke, Steare Thomas, Williams Julie, Wood Lisa, Gaughran Fiona, Johnson Sonia (2021). Implementation of telemental health services before COVID-19: rapid umbrella review of systematic reviews. J Med Internet Res.

[59] Sampson JP, Kolodinsky RW, Greeno BP (2011). Counseling on the information highway: future possibilities and potential problems. Jour of Counseling & Develop.

[60] White M, Dorman S M (2001). Receiving social support online: implications for health education. Health Educ Res.

[61] Dolev-Amit T, Leibovich L, Zilcha-Mano S (2020). Repairing alliance ruptures using supportive techniques in telepsychotherapy during the COVID-19 pandemic. Counselling Psychology Quarterly.

[62] Simon SR, Evans JS, Benjamin A, Delano D, Bates DW (2009). Patients' attitudes toward electronic health information exchange: qualitative study. J Med Internet Res.

[63] Shah SM, Khan RA (2020). Secondary use of electronic health record: opportunities and challenges. IEEE Access.

[64] Staring K (2011). Organizational open source in the Global South [PhD thesis]. University of Oslo.

[65] Kichloo Asim, Albosta Michael, Dettloff Kirk, Wani Farah, El-Amir Zain, Singh Jagmeet, Aljadah Michael, Chakinala Raja Chandra, Kanugula Ashok Kumar, Solanki Shantanu, Chugh Savneek (2020). Telemedicine, the current COVID-19 pandemic and the future: a narrative review and perspectives moving forward in the USA. Fam Med Community Health.

[66] Tyree PT, Lind BK, Lafferty WE (2006). Challenges of using medical insurance claims data for utilization analysis. Am J Med Qual.

[67] Ho Chan WS (2010). Taiwan's healthcare report 2010. EPMA J.

[68] (2010). Health financing challenges and institutional options to move towards universal coverage in Nicaragua. World Health Report.

[69] Lahham Aroub, McDonald Christine F, Mahal Ajay, Lee Annemarie L, Hill Catherine J, Burge Angela T, Cox Narelle S, Moore Rosemary, Nicolson Caroline, O'Halloran Paul, Gillies Rebecca, Holland Anne E (2018). Home-based pulmonary rehabilitation for people with COPD: A qualitative study reporting the patient perspective. Chron Respir Dis.

[70] Ensor T, Cooper Stephanie (2004). Overcoming barriers to health service access: influencing the demand side. Health Policy Plan.

[71] Bhatt SP, Patel SB, Anderson EM, Baugh D, Givens T, Schumann C, Sanders JG, Windham ST, Cutter GR, Dransfield MT (2019). Video telehealth pulmonary rehabilitation intervention in chronic obstructive pulmonary disease reduces 30-day readmissions. Am J Respir Crit Care Med.

[72] Bhatt SP, Baugh D, Hitchcock J, Kim Y, Cutter G, Aban I, Dransfield MT (2022). Video telehealth pulmonary rehabilitation for chronic obstructive pulmonary disease is associated with clinical improvement similar to center-based pulmonary rehabilitation. Ann Am Thorac Soc.

[73] Brownstein JS, Freifeld CC, Madoff LC (2009). Digital disease detection--harnessing the web for public health surveillance. N Engl J Med.

[74] Lavin B, Dormond C, Scantlebury MH, Frouin P, Brodie MJ (2020). Bridging the healthcare gap: Building the case for epilepsy virtual clinics in the current healthcare environment. Epilepsy Behav.

[75] Delitto A, Erhard R E, Bowling R W (1995). A treatment-based classification approach to low back syndrome: identifying and staging patients for conservative treatment. Phys Ther.

